# Development of an Automated Optical Inspection System for Rapidly and Precisely Measuring Dimensions of Embedded Microchannel Structures in Transparent Bonded Chips

**DOI:** 10.3390/s21030698

**Published:** 2021-01-20

**Authors:** Pin-Chuan Chen, Ya-Ting Lin, Chi-Minh Truong, Pai-Shan Chen, Huihua-Kenny Chiang

**Affiliations:** 1 Department of Mechanical Engineering, National Taiwan University of Science and Technology, Taipei 106, Taiwan; m10703116@mail.ntust.edu.tw (Y.-T.L.); Cmtruongb10635001@gmail.com (C.-M.T.); 2 High Speed 3D Printing Research Center, National Taiwan University of Science and Technology, Taipei 106, Taiwan; 3 Graduate Institute of Toxicology, College of Medicine, National Taiwan University, Taipei 100, Taiwan; paishanchen@ntu.edu.tw; 4 Institute of Biomedical Engineering, National Yang-Ming University, Taipei 112, Taiwan

**Keywords:** automated optical inspection (AOI), inspection of bonded microfluidic chip, machine vision, microfluidics

## Abstract

This study aimed to develop an automated optical inspection (AOI) system that can rapidly and precisely measure the dimensions of microchannels embedded inside a transparent polymeric substrate, and can eventually be used on the production line of a factory. The AOI system is constructed based on Snell’s law. The concept holds that, when light travels through two transparent media (air and the microfluidic chip transparent material), by capturing the parallel refracted light from a light source that went through the microchannel using a camera with a telecentric lens, the image can be analyzed using formulas derived from Snell’s law to measure the dimensions of the microchannel cross-section. Through the NI LabVIEW 2018 SP1 programming interface, we programmed this system to automatically analyze the captured image and acquire all the needed data. The system then processes these data using custom-developed formulas to calculate the height and width measurements of the microchannel cross-sections and presents the results on the human–machine interface (HMI). In this study, a single and straight microchannel with a cross-sectional area of 300 μm × 300 μm and length of 44 mm was micromachined and sealed with another polymeric substrate by a solvent bonding method for experimentations. With this system, 45 cross-sectional areas along the straight microchannel were measured within 20 s, and experiment results showed that the average measured error was less than 2%.

## 1. Introduction

Based on a report and data from a US market research firm in May 2019 [1], the global biochip market will grow at a rate of 11.7% to reach a record of 23.97 billion USD by 2026. The major factors causing the increase in research and investment in the biochip market are immune diseases, cancers, viral infections, and chronic diseases in the Asia–Pacific region. Among the wide range of biochips, the development and manufacture of microfluidic chips is the major focus of many governments, industries, and academic organizations. The development of microfluidic chips began in the 1980s and 1990s. Today, the rise of the semiconductor industry has led to the development of micro-electro-mechanical-systems (MEMS) [2]. This advancement allows us to integrate many microdevices, such as microelectronics, a microsensor, microactuator, micropump, microvalve, etc., into microfluidic chips and use them for disease detection [3], new drug development [4], genetic testing [5], and many other applications.

The purpose of a microfluidic chip is to perform a series of chemical/biochemical processes with a small amount of liquid sample. The benefits of those microfluidic chips are reduced processing times, decreased labor costs, and the provision of accurate, molecular level detection [6]. Because of the potential of such a miniaturized and portable device for various applications, the consumption rate for disposable microfluidic chips is significantly increasing, which leads to the need for the mass production of these microfluidic devices. From the perspective of industrial mass-production techniques for any type of product, injection molding is the major and mature process that is used with various polymeric materials. Polymeric microfluidic chips have become mainstream substrate materials due to their low costs, mechanical and chemical characteristics, excellent optical properties, fabricability, machinability, biocompatibility, etc. [7]. The microchannel can be fabricated with a metal mold insert via injection molding, and the microchannel is sealed with bonding techniques, such as thermal bonding [8], adhesive bonding [9], and solvent bonding [10]. However, these bonding techniques might change the dimension of the microchannels, and those dimensional changes might influence the fluid dynamics inside the microchannel and thereby affect the final performance. Therefore, an inspection system that is capable of providing highly accurate, noncontact, nondestructive, fully automated, and instant results is preferred and crucial to production processes of microfluidics industries.

Metrology is one of the most important aspects when it comes to manufacturing a microfluidic chip, because the quality and functionality of these microfluidic devices mainly depend on the correct dimension of the microchannel embedded inside the chip. To ensure the correct dimension of the microchannel, optical methods are usually used for their noncontact inspecting ability. These measurement techniques are interferometric microscopy [11], confocal fluorescence microscopy (CFM) (5–6% uncertainty) [12], optical coherence tomography (OCT) (4% uncertainty) [12], laser interferometry (0.3% uncertainty) [13], etc. The automated optical inspection (AOI) system introduced herein is capable of measuring nine microchannel cross-sections in one picture frame, fast and rapid inspection processes and adapting to the microchannel position. This inspection concept can be developed to automatically and rapidly measure more complex microfluidic structures in the future.

In this study, an automated optical inspection (AOI) system is developed with the aim to rapidly inspect the dimensional accuracy of a bonded microchannel by using a machine vision technique with programmed algorithms to automatize the system. This program helps the system to automatically control the camera position, measurements, calculations, and data presentation. After pushing AUTO START, the camera will move to the inspection area and start taking pictures. These pictures are inspected by the machine vision program, in which the width and height along the microchannel length are calculated using custom-developed formulas. Specifically, these custom-developed formulas were derived from Snell’s law and geometrical angle analysis. 

In the experiments, a single, straight microchannel with a cross-sectional area of 300 μm × 300 μm and length of 44 mm was microfabricated and used for experiments. This paper focuses on the construct, program, and algorithms of the AOI system. After that, we explain the experiment method and result and provide discussion and more information about the system’s performance.

## 2. Microfabrication of Microfluidic Device and Concept of AOI System

### 2.1. Microfabrication of Microfluidic Chip

**Figure 1:**
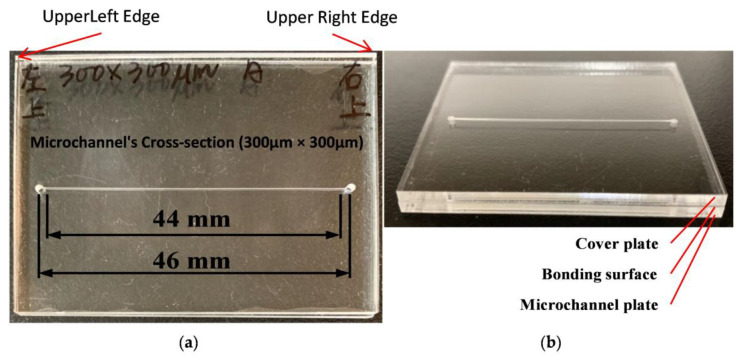
(**a**) Microchannel and entrance and exit injection ports with a total length of 46 mm and a microchannel length of 44 mm; (**b**) Bonded polymethylmethacrylate (PMMA) microfluidic chip after solvent bonding with layers description.

In previous studies, the type of polymers commonly used for microfluidic chips are polymethylmethacrylate (PMMA) [14], polydimethylsiloxane (PDMS) [15], polyethylene terephthalate (PETE) [16], polycarbonate (PC) [17], and polystyrene (polystyrene, PS) [18]. In this study, the microfluidic chip is made of polymethylmethacrylate (PMMA); the structure and dimensions of the microchannel chip are shown in Figure 1. Figure 1a shows the overall view of the microfluidic chip, and Figure 1b shows the bonded layers of the microfluidic chips.

A micromilling machine was used to create the trench for the channel on one PMMA plate. Two holes were drilled on both ends of the trench to form entrance and exit injection ports. The microchannel cross-section is a square (width 300 μm, depth 300 μm) and the flow path is a straight line with a total length of 46 mm, or 44 mm excluding the entrance and exit injection ports. The micromilled substrate was then bonded to another 1 mm thick, flat PMMA plate. The bonding method was reported in a previous study [10], in which a solvent bonding method with an ethanol solution was used. Solvent bonding is the use of specific organic solvents to break down the polymer chains on a surface and diffuse them throughout that surface. A spin coating machine was employed to spread the ethanol solution evenly on the bonding surfaces of the two plates. Next, the two PMMA plates were attached and placed in a UV light machine, in which the UV exposure provided energy for the relinking of the polymer chains. The polymer chains of these two PMMA plates were relinked to form a permanent bond.

### 2.2. AOI System Design and Concept

**Figure 2:**
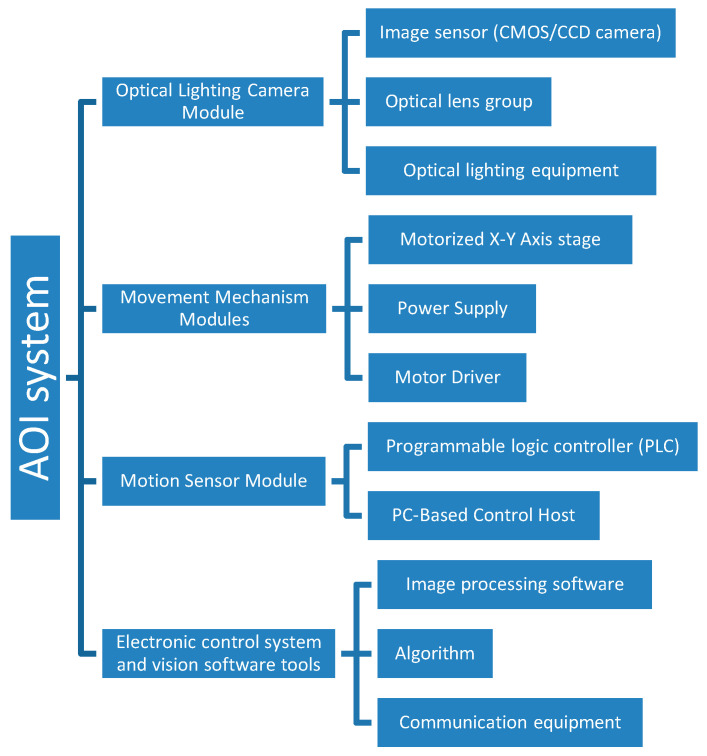
Schematic of the automated optical inspection (AOI) system components and concepts with 4 main modules: optical lighting camera module, a movement mechanism module, a motion sensor module, and an electronic control system and vision software.

The basic structure of the automatic optical inspection system is shown in Figure 2, which includes an optical lighting camera module, a movement mechanism module, a motion sensor module, and an electronic control system and vision software. The automated optical inspection system for transparent microfluidic devices was developed based on the following stages.

#### 2.2.1. AOI System Setting

**Figure 3:**
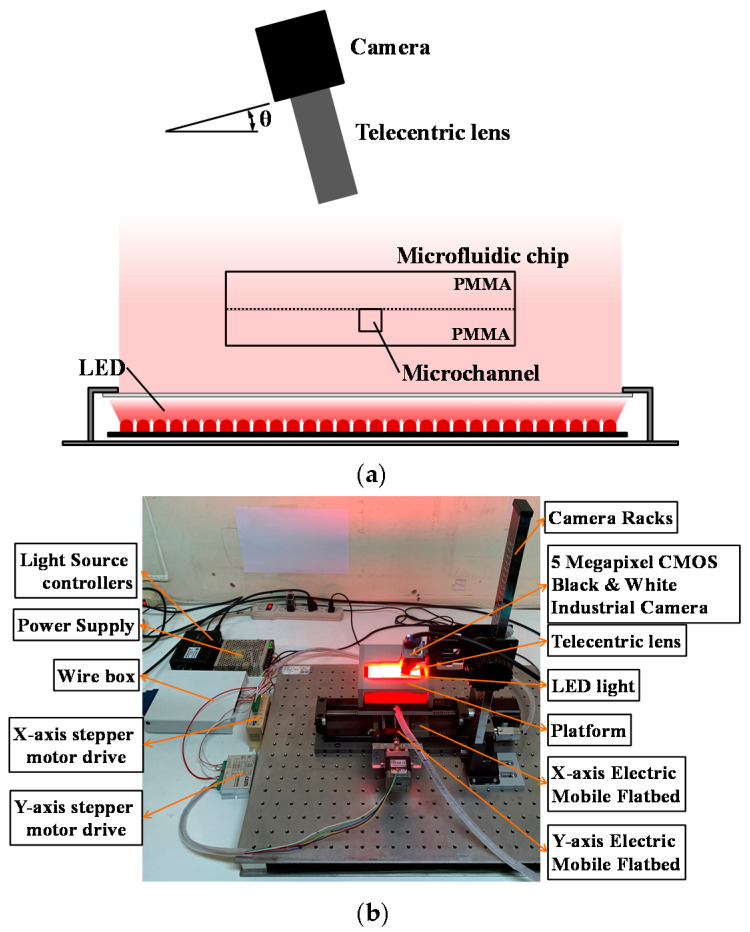
(**a**) Camera, microfluidic chip, and LED light positioning; (**b**) system components assembly.

A CMOS black-and-white industrial camera with a 1x telecentric lens was used to monitor the chip. The camera was rotated at an angle θ to the vertical direction of the chip surface. An LED light source was designed to lie parallel under the chip surface, and it was used to provide good lighting conditions to capture images. Figure 3a shows the schematic for the camera angle and LED light setting. Two electrical stepper motors were used to control an X–Y axis horizontal platform. The microfluidic chip and the light source were subsequently fixed to the top of the X–Y platform to control the chip movement and position. Figure 3b shows the system placement and assembly.

**Figure 4:**
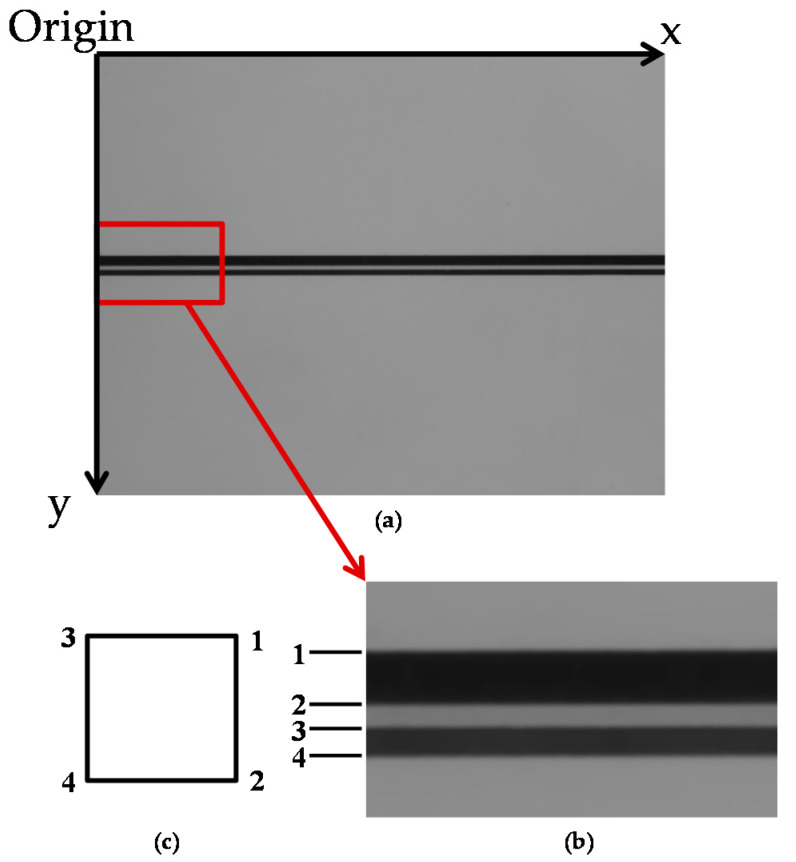
(**a**) Image of the microchannel after being processed and filtered by the NI Vision Assistant software. The image has been pixelated to create an X–Y pixel coordinate system. The X–Y axes showing the positive direction of the coordinates; (**b**) the enlarged image of the microchannel part to have a closer look at the edges of the cross-section in the microchannel; the edges are labeled as 1, 2, 3, and 4 corresponding to the edges in Figure 4c; (**c**) the square cross-section of the microchannel with 4 edges labeled as 1, 2, 3 and 4.

NI Vision Assistant 2018 SP1 was used to process the image and measure the details. The software can sharpen the important features and fade out the unwanted defects. After processing the image, the system creates an X–Y coordinate system using pixels as the unit of measurement with the positive dimensions described in Figure 4. Figure 4 explains the X–Y coordinate system in a microchannel section image (taken at a 35° camera angle). This AOI system was built based on the NI LabVIEW programming platform, which includes image acquisition, image processing, algorithms, etc., that can control chip movement and the ability to process a wide range of data sources. After the image is captured, the data are calculated using formulas based on Snell’s law. The measurement data are shown on the human–machine interface. The microfluidic chips used in this experiment are made of PMMA, which has a refractive index of 1.49 [19]. The measurement method is established in a single area (the field of view of the camera); the light source and the image of the microfluidic channel during the recording process are observed and adjusted to optimize the lighting condition and angle of view. When the camera is rotated at an angle θ, the four corners of the rectangular cross-section (1, 2, 3, and 4) in the microfluidic channel can be acquired simultaneously, as shown in Figure 4b,c, which corresponds to the width and height of the microchannel.

#### 2.2.2. System Design and Algorithms

Snell’s law states that when light passes through two different transparent materials, if the refractive index of the two materials differs, then refraction can occur. Equation (1) by Snell’s law describes the relationship between the incident line and the refracted line, and the vertical dimension of the refracting surface.

*n*_1_ sin (*θ*_1_) = *n*_2_ sin (*θ*_2_), (1)

where *n*_1_ is the refractive index of medium 1, *n*_2_ is the refractive index of medium 2, *θ*_1_ is the incident angle, and *θ*_2_ is the refractive angle.

**Figure 5:**
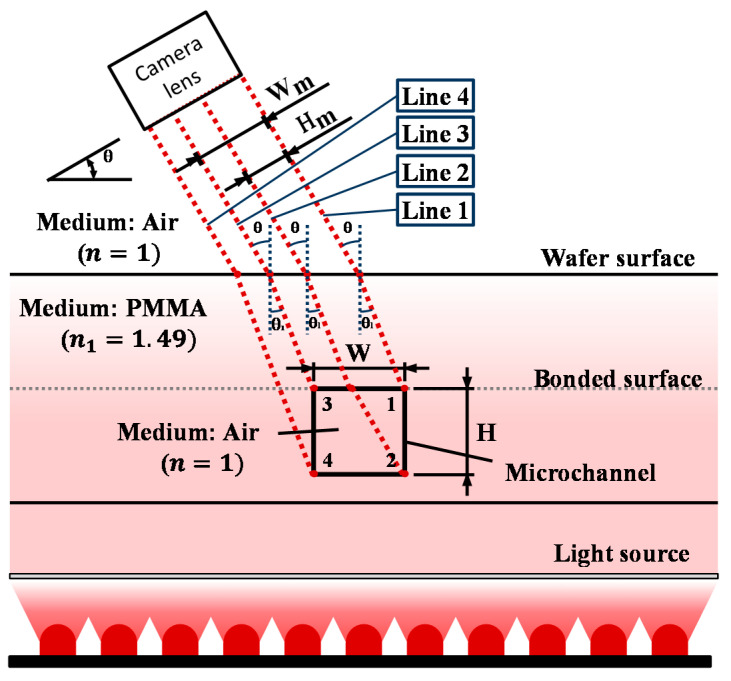
The light path from the edges of the microchannel cross-section through the system environments to the camera.

By rotating the camera at a certain angle, the light received by the telecentric lens will allow the camera to have a full view of all the features in the microchannel. Figure 5 shows the light path of the microchannel features in the system using Snell’s law. The machine lens receives light to photograph the schematic features of the channel. From Figure 5, we understand that at a camera angle setting larger than 45°, the edges of the microchannel can overlap one another in the picture, and if the camera angle is at 0°, the camera view cannot receive the data to calculate the microchannel height from the image. The appropriate camera angle setting should be greater than 0° for both Wm and Hm to be visible, and smaller than 45° to avoid overlapping the edges of the channel.

The simulation shown in Figure 5 was performed with the refractive index of air *n*_1_ = 1 and the refractive index of PMMA set to *n*_2_ = 1.49. Wm and Hm are the data that the system can obtain from the microchannel image. W is the actual width of the microchannel, and H is the actual height of the microchannel wall. To measure the height of the microchannel, line 2 has passed through the air medium inside the microchannel, so it is refracted at a wider angle and enters the PMMA medium again at the bonded surface, in a position further from line 1. The line started from edge 4 only travels in the PMMA, so it arrived at the bonded surface at a smaller angle. This explains why lines 1 and 2 are further apart than lines 3 and 4. In this case, using the distance from line 1 to line 2 to calculate the microchannel height is preferred, because the formula can be applied with the same angle as the formula used to calculate the width. This assures consistency for the formulas.

After the system collects the picture, the image can be analyzed by the following Formulas (2)–(4). In Figure 5, the keys to measuring W and H are lines 1, 2, and 3, and the simulation results show that lines 1, 2, and 3 are parallel to each other. They have the same path length inside the PMMA substrate, regardless of the changes of *θ* and *θ*_1_. The distance between the two paths will not change, so the lengths of Wm and Hm in the picture are independent of the horizontal position of the camera. From the relationships between Wm and W and between Hm and H shown above, the algorithms to calculate the width and height were built using trigonometric functions.

**Figure 6:**
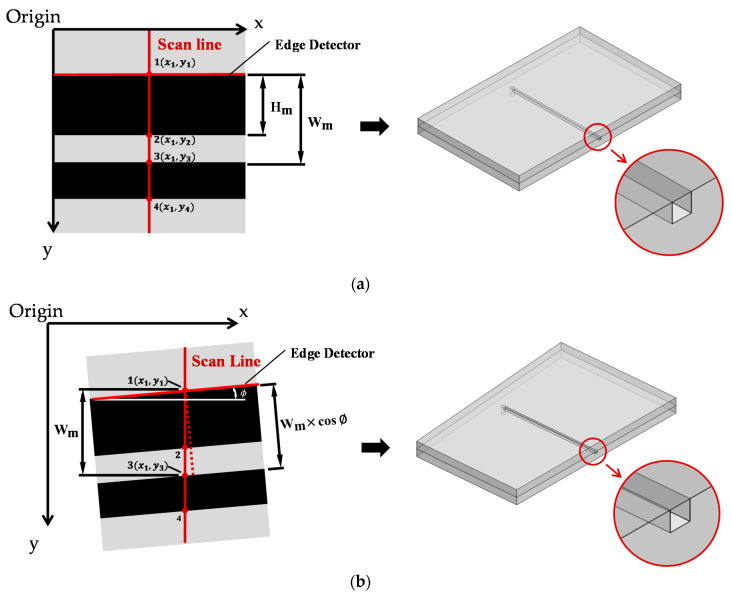
(**a**) Scan line, edge detector, and the cross-section of the channel at the scan line (when the microchannel is perpendicular to the scan line); (**b**) scan line, edge detector and the cross-section of the channel at the scan line (when the microchannel is not perpendicular to the scan line).

The following is an explanation of how to obtain Wm and Hm lengths using the microfluidic feature coordinates. Figure 6a,b demonstrates the principles of using the scan line, the straight edge detecting system, and the comparison of the cross-sections in two cases. The first case is if the microchannel is perpendicular to the scan line, while the second case is when the microchannel is tilted at a *ϕ* angle from the perpendicular position.

For both cases in Figure 6a,b, the vertical scanline acts as the region of interest and it receives the location coordinates of the points 1, 2, 3, and 4 based on the origin point. The coordinates of points 1, 2, 3, and 4 correspond to the microfluidic cross-section at edges 1, 2, 3, and 4, respectively.

A straight edge detector is used to find the straight-line features of the microchannel from top to bottom of the image. Through this function, the system can identify the tilt angle *ϕ* of the channel. Next, the two sample microchannel cross-sections are exposed at the scan line positions to be examined with a microscope (the right-side figures of Figure 6a,b).

According to Figure 6a, to calculate Wm, the y-coordinate of point 3 is used to subtract the y-coordinate of point 1. The unit of this length is in pixels, so the conversion to the μm unit is needed by a scale factor (please refer to Supplementary Materials Figure S1 and Table S1). The length of Wm can be obtained by converting it to μm and then by substituting Wm into Equation (2), we can obtain the microchannel width (W).

(2) $$\frac{\mathrm{Wm}}{\mathrm{Cos}\theta }=W$$

To calculate the height of the microfluidic channel, we use the y-coordinate of point 2 to subtract the y-coordinate of point 1; the length of Hm can be obtained by converting the scale factor and then substituting it into Equation (3).

(3) $$\frac{\mathrm{Hm}}{\mathrm{Sin}\theta }=H$$

Above is the algorithm established under the condition that the chip features are perfectly horizontal (Figure 6a). When the microchannel is not perfectly perpendicular to the scan line, it was found that the wrong width is recorded (Figure 6b). However, the height of the cross-section can be precisely measured, and the height value does not depend on the *ϕ* angle of the channel. Therefore, a revised formula is needed to calculate the width of the microchannel when the microchannel is not perfectly perpendicular to the scan line.

Equation (4) is used when the microchannel is not fully horizontal to calculate the width of the microchannel. The next step determines the width of the microfluidic channel by using trigonometric calculation. In Figure 6b, the value Wm can be calculated by using the y-directional coordinate of point 3 minus the y-directional coordinate of point 1. Next, convert the unit of Wm from pixel to μm, and substitute Wm and *ϕ* into Equation (4) to calculate the correct width.

(4) $$\frac{\mathrm{Wm}\times \mathrm{Cos}\phi }{\mathrm{Cos}\theta }=W$$

The tilt angle *ϕ* is 0 in the case where the chip is not tilted, then Equation (4) is identical to Equation (3).

**Figure 7:**
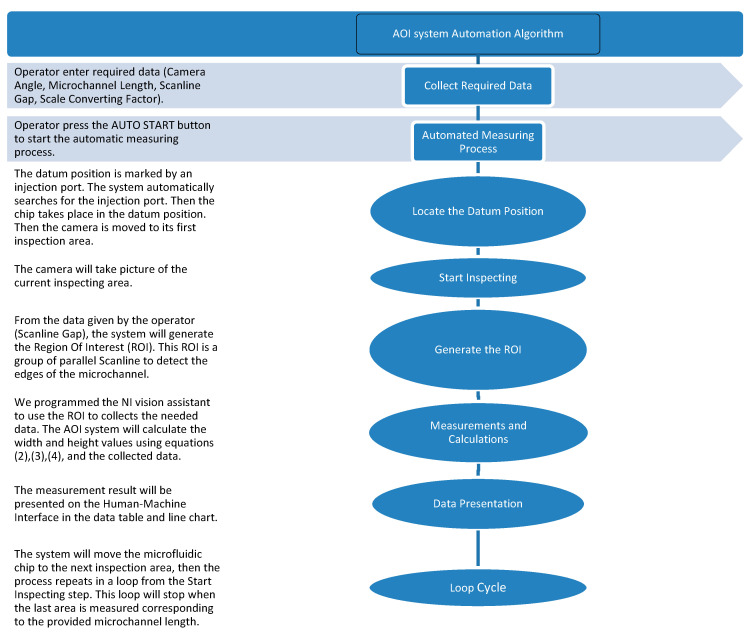
AOI system program concept and steps explanation.

In order to automatize the machine’s hardware and software to perform all the required tasks, a program is developed. Figure 7 explains the basic structure of the program and the operational steps.

## 3. Experiment Procedure

**Figure 8:**
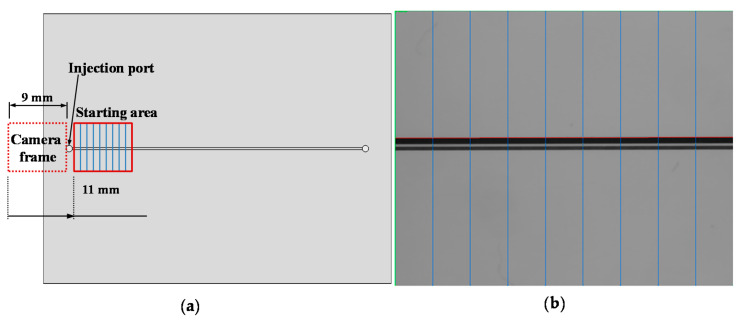
(**a**) Camera datum position distance to the initial starting area of the measurement; (**b**) scanning lines created using NI Vision Assistant.

To conduct the experiment procedure, the AOI system is programmed to work according to the following steps. When started, the system is designed to automatically locate the injection port. This is the first step to locating the starting point of the measurement. Next, move the injection port to the right side of the camera frame (until the part is just outside of the picture frame) as a datum position, and it subsequently moves 11 mm to the first measurement area (Figure 8a). After capturing the features of the starting area, the camera continues the inspection in the next area along the channel. Figure 8b shows that each measuring frame has nine vertical scan lines to inspect Wm and Hm at nine cross-sections of the microchannel in one picture. The gaps between the lines are 1 mm. After measurement and calculation, the data are presented on the human–machine interface (HMI) system (please refer to the Supplementary Materials, Figure S2). Two experiments are carried out to characterize the performance of our developed system, including the camera angle setting experiment and microchannel dimension determination experiment. The calculation of the error is based on the deviation between the system measurements and microscopic measurements for each cross-section. The standard error is then calculated from the errors of the 45 cross-sections to obtain the standard deviation. 

### 3.1. Camera Angle Setting Determination

This experiment was carried out to understand the influence of the camera angle on measurement accuracy to improve detection accuracy. The experiments showed that the contrast at the edges is not clear for accurate detection at low camera setting angles (*θ* = 5°, 10°, 15°), while the contrast at the cross-section edges is higher at a higher camera angle, and the program can detect the points more precisely (please refer to the Supplementary Materials, Table S2). As a result, the microchannel is measured at five different camera-setting angles (*θ* = 20°, 25°, …, 40°) to ensure the influence (image contrast) of the camera setting angle to the final measurement accuracy. 

### 3.2. Microchannel Dimensions Determination Using Microscope

In the following, we illustrate how to measure the microchannel width and height using a microscope. This experiment can determine the actual dimension of the microfluidic chip, which can be used to calculate the errors from the results obtained from the developed AOI system. 

To determine the width of the microchannel, we placed the chip horizontally on the stage of the microscope, then started measuring at 2 mm away from the left edge of the injecting port along the channel, 1mm at a time. Figure 8 shows the top view of the chips and describes the measuring process of the width of the microchannel. 

**Figure 9:**
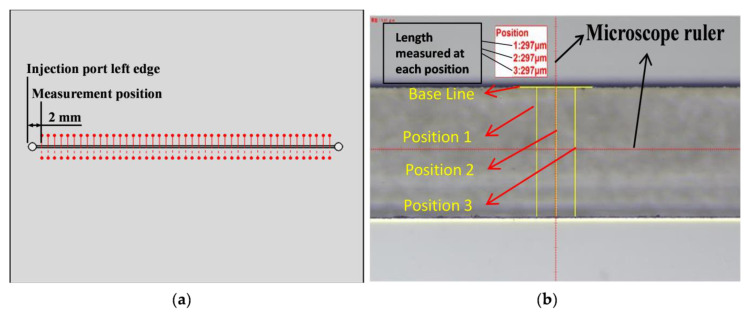
(**a**) Start position for measuring microchannel width with a microscope (top view); (**b**) microscope measurement (top view).

To use the microscope, a base line has to be drawn out first. In Figure 9b, the base line is the horizontal yellow line, and the purpose of this line is to represent the direction of measurement which is along microchannel length. After this, along the measurement direction, the user can set up measurement dimensions, which are the vertical yellow lines starting from the base line to the point of interest. The microscope reported the data on the screen.

**Figure 10:**
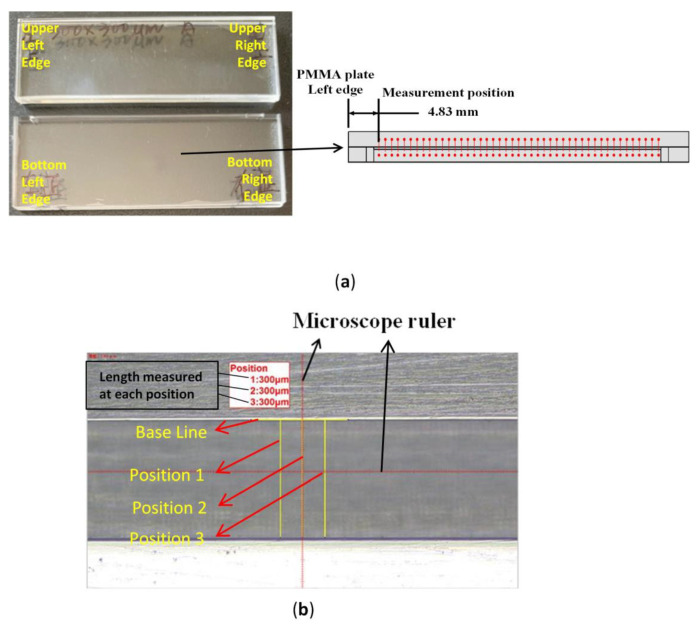
(**a**) Microfluidic chip piece after being cut and the schematic of the start position for measuring the microchannel height on the exposed microchannel area after the cut; (**b**) microscope measurement (side view).

To determine the height of the microchannel, we took a closer look at the sidewall of the microchannel. The chip needs to be cut right in the middle of the microchannel. A utility knife was used to create a line of weak points on the centerline of the microchannel. We then applied force to break the weakened points. The exposed surface was polished with sandpaper on a spinning grinder. Figure 10a shows the cutting result of the microfluidic chip (side view) and the microchannel is exposed for measuring (Figure 10b). After the chip was cut and polished, the piece of the chip with the exposed microchannel was placed vertically on the microscope stage for measurement.

## 4. Result and Discussion 

**Figure 11:**
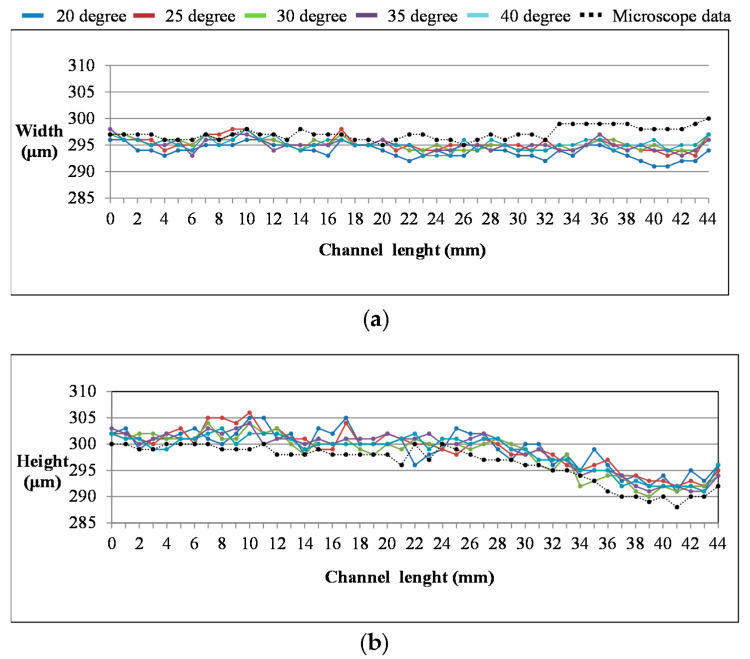
The comparison between the experimental data of 45 microchannel cross-sections measured by the AOI system and experimental data with a microscope at different camera setting angles; (**a**) width of the microchannel; (**b**) height of the microchannel.

Figure 11 shows the experimental measurement of 45 microfluidic cross-sections obtained from different camera mounting angles in comparison with the 45 microfluidic cross-sectional scales measured by the microscope, respectively. Figure 11a shows the measured width, while Figure 11b shows the measured height. 

As we can observe from Figure 11a,b, there were deviations between the AOI measurement and microscope measurement, which possibly came from the light scattering at the edges of the microchannel, the refractive index of the transparent material, and the sharpness of the microchannel edges. Another observation from Figure 11 is the influence of the camera angle setting on the measurement accuracy, and Table 1 shows the results. In Table 1, the average error and standard deviation of the camera setting angle at 20° and 25° are slightly larger than 30°, 35°, and 40°. Therefore, in this study, 30°, 35°, and 40° are used to set up the camera, with the accuracies of width and height measurements being less than 2%. Moreover, the data line for each angle of measurement consistently resembles the microscope data along the whole length of the microchannel, especially at 30°, 35°, and 40°. The experiment result shows that this system is not only accurate on average, but that the standard deviation is also less than 0.7%, and the AOI system is capable of performing quality control and inspections for the production of microfluidic chips [20]. The future development for this AOI system will focus on inspecting microfluidic chips with complex structures and different transparent materials, as the demand for microfluidic chips increases. 

## 5. Conclusions

In recent years, the market demand for real-time diagnostic equipment has increased dramatically, and research on microfluidic manufacturing technology and applications has also flourished. The advantages of microfluidic chips are their ability to be mass-produced and manufactured rapidly. To avoid pollution caused by secondary use, disposable microfluidic chips have been developed, which has led to an increase in production volume and has entered the stage of commercial mass production. The focus of this research is to establish a microscale measurement method to inspect the dimensions of transparent bonded microfluidic chips with an AOI system. The AOI system is built to operate using machine vision technology and custom-developed mathematical equation. The machine core is running on an integrated program. The integrated program can perform a variety of tasks including image processing, feature computation, control chip movement, and measurement data management. The human–machine interface is designed to allow the operator to set basic parameters and control the whole system. With the simple push of a button, the system will automatically measure the cross-sections of the entire microchannel, and the results will be shown on the HMI interface. From the experiment results, it is apparent that this developed system can finish the measurement of 45 cross-sections of a straight microchannel with errors of less than 2% within 20 s. The results demonstrate that this system has high accuracy, high repeatability, and high inspection speed, with a noncontact, nondestructive inspecting method for microchannels embedded inside the bonded polymeric chips. With an average standard deviation of less than 0.7%, this AOI system is qualified to inspect microchannel structures and detect unwanted defects on the mass production line.

## Figures and Tables

**Table 1:** Data errors between the AOI system and the microscope.

Degree	Width		Height	
	Average Error (%)	Standard Deviation (%)	Average Error (%)	Standard Deviation (%)
20	2.35	0.62	2.35	0.58
25	2.01	0.53	2.34	0.58
30	1.67	0.51	1.68	0.44
35	1.68	0.51	1.69	0.45
40	1.67	0.46	1.69	0.38

## Data Availability

The data presented in this study are available on request from the corresponding author. The data are not publicly available due to further study will be carried out using the same data.

## Supplementary Materials

The following are available online at https://www.mdpi.com/1424-8220/21/3/698/s1, Figure S1. (a) Standardized ruler; (b) Standardized ruler image from 0 to 8 mm taken by CMOS black-and-white industrial camera; Figure S2. Human-Machine Interface (HMI); Table S1. Standardized ruler length in pixel and Scale Converting Factor; Table S2: Camera view in different setting angle.
