# Supplementary material for: Development of an Automated Optical Inspection System for Rapidly and Precisely Measuring Dimensions of Embedded Microchannel Structures in Transparent Bonded Chips
Source: Sensors (Basel). 2021 Jan 20;21(3):698. doi: 10.3390/s21030698 (PMC7864200; doi:10.3390/s21030698)
Supplement: Supplementary file 1 [file sensors-21-00698-s001.pdf]

Supplementary File

Development of an Automated Optical Inspection System for Rapidly and Precisely Measuring Dimensions of Embedded Microchannel Structures in Transparent Bonded Chips

Pin-Chuan Chen [1,2,*], Ya-Ting Lin [1], Chi-Minh Truong [1], Pai-Shan Chen [3] and Huihua-Kenny Chiang [4,*]

1. Scale Converting Factor Determination (Pixel to μm)

The purpose of this section is to determine the Scale Converting Factor from pixel to μm. First, the camera is set perfectly perpendicular to the chip holding base. Then a standardized ruler is placed on the holding X-Y base position. Figure S1 shows the Standardized ruler and its image through the camera.

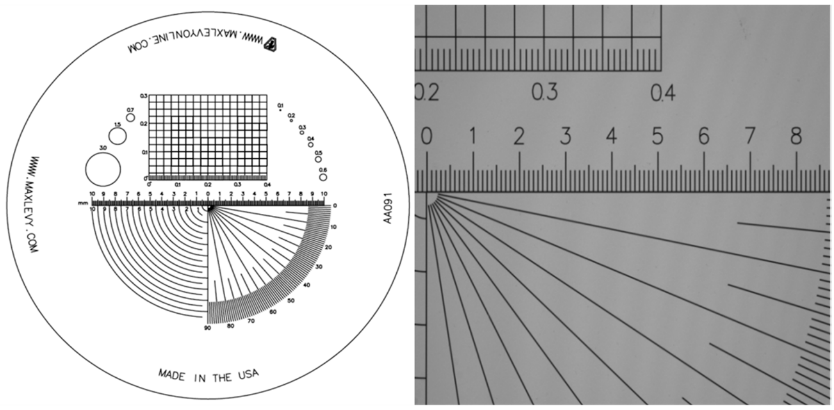

(**a**) (**b**)

Figure S1. (**a**) Standardized ruler; (**b**) Standardized ruler image from 0 to 8 mm taken by CMOS black-and-white industrial camera.

Through Figure S1(**b**) we can observe the ruler. Using NI vision Assistant the distance from point 0 to 8 mm can be determined in the pixel scale. We collect the distance from 10 pictures similar to the above to determine the length of 8 mm (8000 μm) in pixel scale.

The Scale Converting Factor from the pixel unit to μm is determined by dividing 8000 μm by the corresponding average pixel value in the following equation (1).

$$\text{Scale Converting Factor } = \frac{\text{Lenght in } \mu\text{m}}{\text{Lenght in pixel}} = \frac{8000}{2175.92} = 3.68 \left(\frac{\mu\text{m}}{\text{pixel}}\right) \quad (1)$$

Table S1. Standardized ruler length in pixel and Scale Converting Factor

Measurement 1	2175.92 Pixels
Measurement 2	2175.92 Pixels
Measurement 3	2175.93 Pixels
Measurement 4	2175.90 Pixels
Measurement 5	2175.92 Pixels
Measurement 6	2175.96 Pixels
Measurement 7	2175.91 Pixels
Measurement 8	2175.90 Pixels
Measurement 9	2175.93 Pixels
Measurement 10	2175.95 Pixels
Average	2175.92 Pixels

Real length (μm)	8000 μm
Scale converting factor	3.68 μm/pixel

Table S2: Camera view in different setting angle

Camera angle (Degree)	View	Camera angle (Degree)	View
0	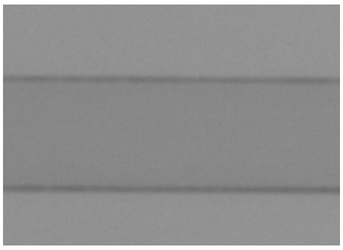	25	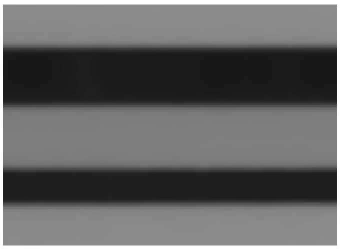
5	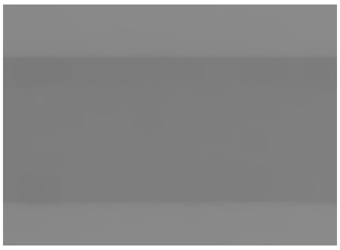	30	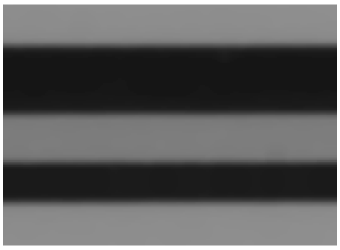
10	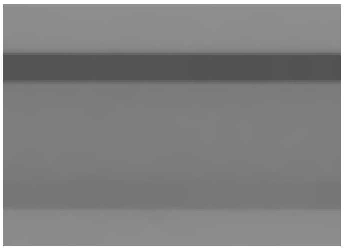	35	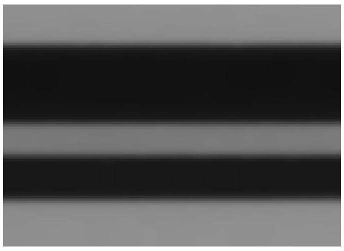
15	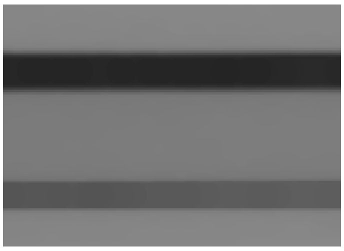	40	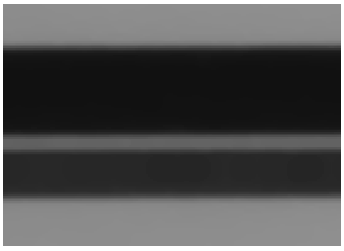
20	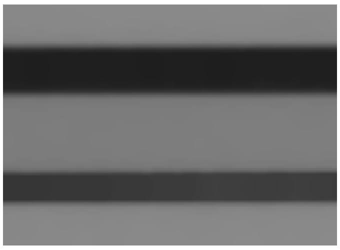	45	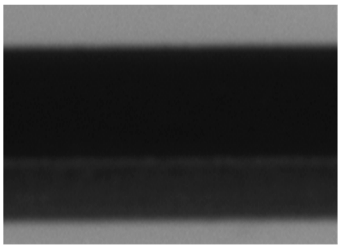

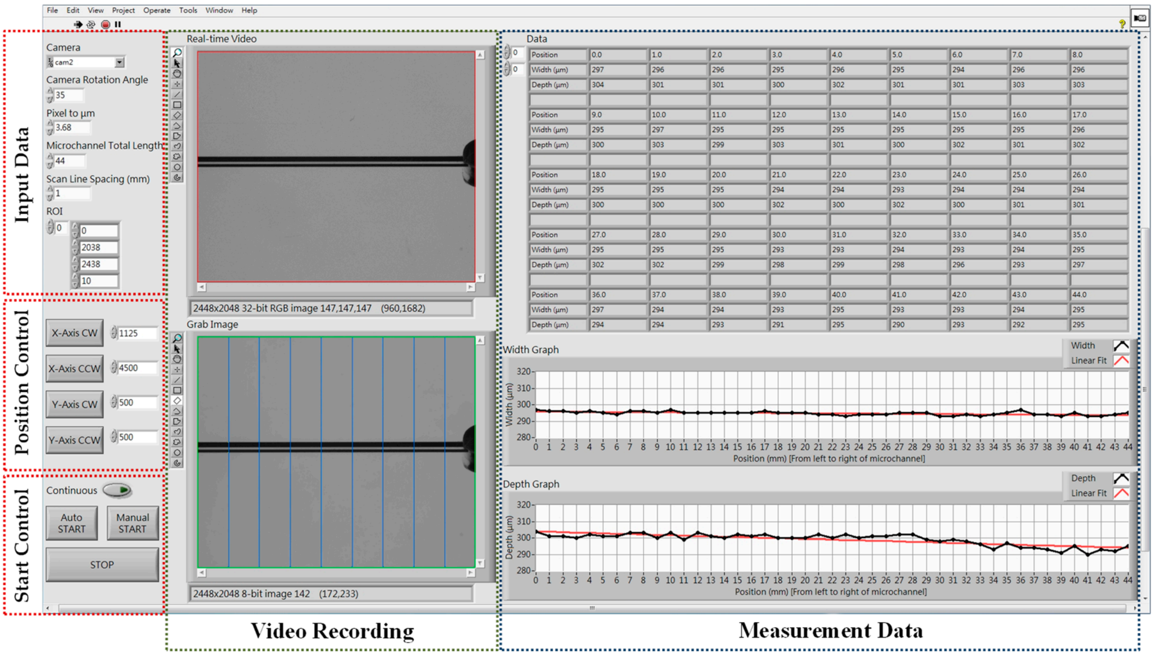

Figure S2. Human-Machine Interface (HMI).
